# Supplementary material for: Quorum Sensing in Halorubrum saccharovorum Facilitates Cross-Domain Signaling between Archaea and Bacteria
Source: Microorganisms. 2023 May 12;11(5):1271. doi: 10.3390/microorganisms11051271 (PMC10223598; doi:10.3390/microorganisms11051271)
Supplement: Supplementary file 1 [file microorganisms-11-01271-s001.zip › microorganisms-2244274-supplementary.pdf]

microorganisms

Supplementary Data

Investigating protease activity of H. saccharovorum CSM52 using azocasein assay

Samples of CSM52 were taken along the growth curve and centrifuged at 12,000 g for 10 min at 4 °C to obtain cell-free supernatant for the determination of protease activity as per the protocol of Paggi *et al.* (2003) [25]. This involved, 100 µL of cell-free supernatant added to 50 µL of azocasein (5 mg/mL), along with 50 µL of Tris HCl (1 M pH 8.5), and 300 µL of NaCl (2.6 M) in a final volume of 500 µL. The mixture was vortexed and incubated at 50 °C for 16 H at 800 rpm. Following incubation, 500 µL of cold 10% TCA was added to stop the reaction, and assay tubes were placed on ice for 30 min. Samples were centrifuged at 3,000 g for 10 min, and the acid-soluble products in the supernatant were added to a multi-well plate and measured at 335 nm using a BMG FLUOstar Optima Fluorescence plate reader (BMG Labtech Ltd, Aylesbury, UK).

Determining the presence of halocins in H. saccharovorum CSM52 using a modified agar overlay assay

The detection of halocins was done based on a modified version of the agar overlay assay. *Hfx volcanii* DS2 was used to create an archaeal lawn, by adding 2 mL of culture between OD_{550} 0.6 and 0.8 into a sterile petri dish and adding 10 mL of P20 and 1% soft agar media held at 55 °C. Plates were swirled to ensure homogeneity, and then left to solidify. 2 mL of *H. saccharovorum* CSM52 culture was centrifuged at 12, 000 g for 2 min every 7 days during incubation at both 37 °C and 45 °C. 20 µL of cell-free supernatants were spotted onto the freshly prepared agar lawns and incubated at 37 °C. Following incubation, plates were observed for inhibition after 24, 48, and 72 H of growth. The diameters of the zones were recorded when the zones reached maximal size. 20 µL of uninoculated media were also spotted on a lawn plate to serve as a negative control.

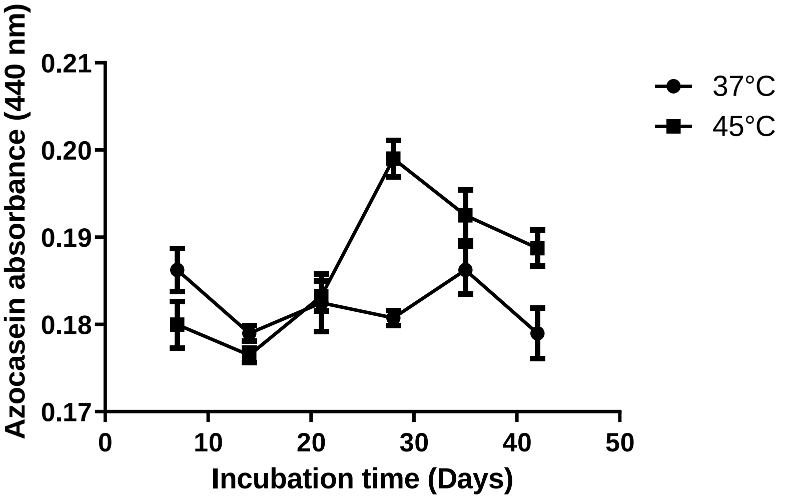

Supplementary Figure S1: Extracellular protease activity profile of *H. saccharovorum* CSM52. Proteolytic activity was measured by the determination of azocasein digestion by cell-free supernatant from CMS52. The supernatant was taken from the culture at 7-day intervals with incubation at both 37 and 45 °C. Values are expressed as the mean and standard deviation of three replicates.

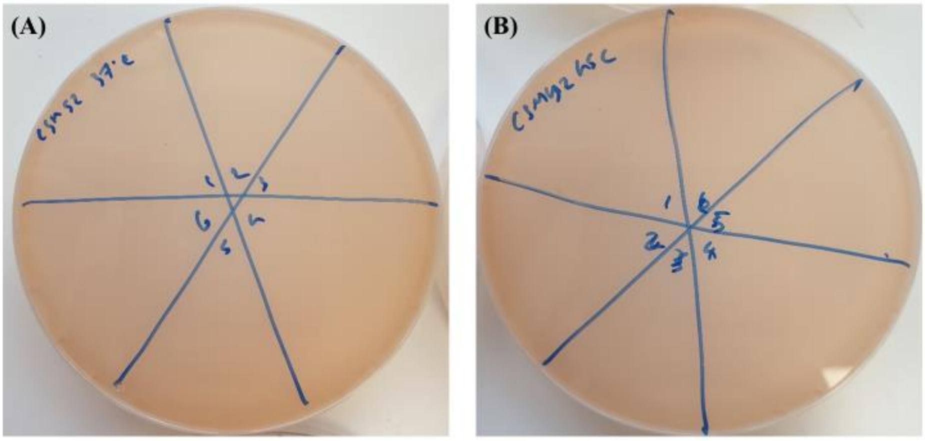

Supplementary Figure S2: (**A**,**B**) *H. saccharovorum* CSM52 was incubated at both 37 and 45 °C, at 7-day intervals, 20 µL of cell free supernatant was spotted onto a lawn of *Hfx. volcanii* DS2, following incubation of 5 days, no zone of inhibition was observed at any time points indicating a lack of competition between the strains.
